# Growth Optimization and Rearing of Mealworm (*Tenebrio molitor* L.) as a Sustainable Food Source

**DOI:** 10.3390/foods12091891

**Published:** 2023-05-04

**Authors:** Kanwal Riaz, Toheed Iqbal, Sarzamin Khan, Amjad Usman, Mariam S. Al-Ghamdi, Ashwag Shami, Rania Ali El Hadi Mohamed, Abdulrahman A. Almadiy, Fahd Mohammed Abd Al Galil, Nawal Abdulaziz Alfuhaid, Nazeer Ahmed, Pravej Alam

**Affiliations:** 1 Department of Entomology, Faculty of Plant Protection, The University of Agriculture, Peshawar 25000, Khyber Pakhtunkhwa, Pakistan; 2 Department of Poultry Science, Faculty of Animal Husbandry and Veterinary Sciences, The University of Agriculture, Peshawar 25000, Khyber Pakhtunkhwa, Pakistan; 3 Department of Biology, Faculty of Applied Sciences, Umm Al-Qura University, Makkah 24381, Saudi Arabia; 4 Department of Biology, College of Sciences, Princess Nourah bint Abdulrahman University, P.O. Box 84428, Riyadh 11671, Saudi Arabia; 5 Department of Biology, Faculty of Arts and Sciences, Najran University, Najran 1988, Saudi Arabia; 6 Department of Biology, Faculty of Science, University of Bisha, P.O. Box 551, Bisha 61922, Saudi Arabia; 7 Department of Biology, College of Science and Humanities, Prince Sattam bin Abdulaziz University, Al-Kharj 11942, Saudi Arabia; 8 Department of Agriculture, University of Swabi, Anbar, Swabi 23561, Khyber Pakhtunkhwa, Pakistan

**Keywords:** fungi, bacteria, mealworm, life parameters, diet supplements

## Abstract

As a sustainable food source for humans, mealworms (*Tenebrio molitor*) have a great deal of potential, due to the fact that they have a very favorable nutritional profile and a low environmental impact. For meal production, feed formulation and optimization are important. The mealworm *Tenebrio molitor* (Coleoptera: Tenebrionidae) is the most consumed insect in the world. Mealworms were given a variety of diets, including wheat bran as constant diet supplemented with different levels of Ospor (*Bacillus clausii*) at 0.002 g, 0.004 g, 0.006 g, and 0.008 g; imutec (*Lacticaseibacillus rhamnosus*) at 0.2 g. 0.4 g, 0.6 g, and 0.8 g; fungi (*Calocybe indica*) at 250 g, 500 g, and 750 g; yeast (*Saccharomyces cerevisiae*) at 50 g, 100 g, and 150 g; and wheat bran (standard diet) were examined in complete randomized design (CRD). Different parameters, i.e., the larval, pupal, and adult weight, size, life span, and nutritional profile of mealworm were studied. When compared with other insect growth promoters, only wheat bran was discovered to be the most efficient. It generated the heaviest and longest larvae at 65.03 mg and 18.32 mm, respectively, as well as pupae weighing 107.55 mg and 19.94 mm, respectively, and adults weighing 87.52 mg and 20.26 mm, respectively. It was also determined that fungi (*C. indica*) and ospor (*B. clausii*) promoted faster larval development than yeast (*S. cerevisiae*) and imutec (*L. rhamnosus*). Larval mortality was also greater in the imutec (*L. rhamnosus*) and yeast (*S. cerevisiae*) diets than the others. No pupal mortality was recorded in all diets. Furthermore, the protein content of *Tenebrio. molitor* raised on a diet including fungi (*C. indica*) was the highest at (375 g), with a content of 68.31%, followed by a concentration of (250 g) with a content of 67.84%, and wheat bran (1 kg) (normal diet) with the lowest content at 58.91%. *T. molitor* larvae fed a diet supplemented with bacterial and fungal had lower fat and ash content than bran-fed *T. molitor* larvae (standard diet). Wheat bran (normal diet) had the highest fat at 16.11%, and ash at 7.71%. Hence, it is concluded that wheat bran alone or diet containing fungi (*C. indica*) and ospor (*B. clausii*) performed better in terms of growth, and these diets and protein content are recommended for the mass rearing of mealworms.

## 1. Introduction

Taking into consideration the fact that insects are becoming more and more accepted as a food source that can be given to humankind, as well as domestic livestock in industrialized countries, then a significant economic change will arise [1]. Moreover, insects have the potential to be used as a means of recycling waste products and resources for the production of highly nutritive diets for many other domesticated animals and for humans.

In many parts of the world, entomophagy, which is the consumption of insects by humans, is practiced [2,3]. While there is increasing research into the viability of edible insects as a food source for humans, entomophagy, or the consumption of insects, remains controversial [4], However, the United Nations’ Food and Agriculture Organization (FAO), has advocated insect farming as an alternative to traditional livestock production [5]. A growing number of food producers are recognizing the economic benefits of using insects as a protein source in both human and animal food production, and this development goes along with the escalating costs of conventional protein sources, such as meat, fish meal, and soybean meal [6]. Moreover, insects are rich in high quality proteins, polyunsaturated fatty acids, dietary fibers, and a variety of micronutrients [7]. Furthermore, to the nutritional benefits, insects have a high feed conversion rate, low environmental footprints, and are significantly less land-dependent than livestock production [5]. 

*Tenebrio molitor* L. (Coleoptera: Tenebrionidae), commonly known as yellow mealworm, is a widespread stored grain pest found in warehouses. In moist, poor conditions, they feed on milled cereals or decaying grain, occasionally infesting maize meal, flour, cake mixes, cereals, meat leftovers, bran, litter from chicken houses, and dead insects; they have been discovered feeding on the droppings of sparrows [8]. Their larva can readily be grown on low-nutrient plant debris. They are made commercially for use as pet food for birds and reptiles and fishing bait [9]. As a result of their high nutritious value and low contamination levels, mealworm larvae are a particularly sustainable source of feed and food [10,11]. Exudates, feces, body parts, and dead insects contaminate plant products [12,13]. They can survive on organic waste [5]. Mealworms can be raised purely on wheat bran, although organic matter, i.e., potatoes, cabbage, and carrots are usually added to their meals to offer essential nutrients [14,15].

Considering the abovementioned importance, the current study was designed to investigate new and organic sources of mealworm food, to give a healthier, natural supplement to the poultry raised locally. The objective of this study was to evaluate the efficacy of different supplements on different growth stages of *T. molitor* L, and to categorize the nutritional profiling of mealworm larvae reared on various levels of supplements.

## 2. Materials and Methods

A study on the assessment of different growth stages of mealworm (*Tenebrio molitor* L.) with different mass-rearing supplements (Ospor^®^, Imutec^®^, Fungi and yeast) was carried out at the Insect Rearing Laboratory, Department of Entomology, The University of Agriculture Peshawar. This is the first time that mealworms have been used in a study to determine the efficacy of dietary supplements.

### 2.1. Establishment of the Stock Culture of Mealworm

Wheat bran was purchased from the nearby market and sterilized in an autoclave (Speedy Autoclave, Model No. HL-340) at 121 °C for 30 min, to make them free from microorganisms. To make wheat bran suitable for larval consumption, it must be sieved through a sieve of mesh size 14, after being passed through the sterilization process. The mealworm colony was cultured in an Environmental Growth Chamber (EGC) at a 25 ± 5 °C temperature, with a relative humidity of 60~65%, and a 10:14 (light/dark) photoperiod.

### 2.2. Diet Preparation

The substrate feed for the mealworms (wheat bran) was prepared from four different food sources, i.e., bacteria spp. (*Ospor* and *Imutec*), fungi, and yeast, as shown in the tables. These food materials were bought from the nearby market. Food with the tradename Ospor^®^ is manufactured by LifeSpo Pharma Co. Ltd., Vietnam Formerly ANA Bio Research & Development JSC Vietnam, it contains spores of poly-antibiotic-resistant Bacillus clausii—2 Billion CFU/5 mL. Imutec^®^ is a tradename of Bifidobacterium + *Lactobacillus rhamnosus* (26 mg + 19 mg) with a dose of 12 billion CFU/1 gm, manufactured and distributed by Searle Pharma Co., Pvt. Ltd., Karachi, Pakistan. To make the diet suitable for larval consumption, it must be ground and sieved through mesh size 14 (1.41 mm) after sterilization. The prepared growth medium was kept in a plastic box (3 cm deep × 11 cm wide × 30 cm long). Fifteen larvae with a diet of 50 g per box were placed in EGC. Potato slices placed at the top of each box served as a source of moisture. Fresh potato slices were provided to growing mealworms on weekly bases until the completion of the study.

### 2.3. Parameters Studied

An experiment was arranged in a completely randomized design (CRD) using the 10 treatments with three replications. Data was collected on larval durations (days), larval weight (mg), and larval size (mm). Pictures of larva were taken with a Nikon SMZ 745 T stereomicroscope mounted with Nikon FSi2 camera. Larval size was measured with the help of an Image j (version 1.8.0) software. Data was also collected on larval mortality (%) and pupal duration (days). The duration of the pupal stage in each diet was calculated separately from the initiation of the pupal stage (last molting stage) until the emergence of adults. Data was further gathered on pupal weight (mg), pupal size (mm), pupal mortality (%), adult longevity (days), adult weight (mg), and adult size (mm). Adults were photographed using a trinocular stereomicroscope Nikon SMZ 745 T mounted with Nikon FSi2 camera. Adult sizes were measured using the software Image j (version 1.8.0). Five individuals from each replication were randomly selected to measure larval, pupal, and adult size. Measurements were made with the software Image j version (1.8.0). Larval measurements were made at the pre-pupal stage (larvae turned to C shape and stopped feeding). Chemical analysis was only carried out at the larval stage, as mentioned under nutritional profile. Pupal measurements were made upon the appearance of dark color spots, indicating mature pupa. The adult size was measured at day 10, when the adult was fully grown, and the mean adult size was calculated. Data were recorded from hatching from egg until 50% of larva was converted to pupa, then the mean larval duration was calculated.

### 2.4. Nutritional Profile

A biochemical analysis of mealworm was carried out at the Veterinary Research Institute (VRI), Peshawar. Mature (late instar) mealworm larvae were dried using a drier at 60 °C for 24 h. After drying, three samples of 10 gm of *T. molitor* of each diet were prepared and subjected for biochemical analysis. Full grown larvae (prepupal stage) were selected for testing nutritional composition (larval length around 25–30 mm).

Crude protein (CP) was analyzed according to the standard Kjeldahl method [16], while crude fat content (EE) was determined as petroleum ether extract [17]. Ash content was determined in a furnace at 600 °C [18].

The protein content was determined using the Kjeldahl method. A total of 2 g dried homogenized sample of *T. molitor* larvae fed on different diets were used for each protein analysis. Samples were mineralized at 420 °C for 105 min. Distillation was carried out for 4 min. The amount of crude protein was calculated by multiplying the content of detected nitrogen by a coefficient of 6.25. The crude protein content was measured three times. The protein content of *T. molitor* in the literature is mainly based on nitrogen content, using the nitrogen-to-protein conversion factor (Kp) of 6.25 generally used for proteins [19,20,21]. 

### 2.5. Percent Larval Mortality

Larval mortality was determined by counting the number of dead larvae among the total number of larvae in each treatment. The larval mortality was converted into percentage by using the formula:

(1) $$Larval\;mortality\;\%=\frac{Dead\;larvae}{Total\;no.\;of\;larvae\;}\times 100$$

### 2.6. Percent Pupal Mortality

The mortality of pupae in a diet was determined by counting the dead pupae (in numbers) from the total number of pupae. Percent pupal mortality was calculated by using the following formula:

(2) $$\%\;Pupal\;mortality=\frac{Total\;no.\;of\;dead\;pupae}{Total\;no.\;of\;pupae}\times 100$$

## 3. Statistical Analysis

The data were submitted to tests of normality of errors and homogeneity of variances. Subsequently, an analysis of variance was performed using the *chi* test (*p* < 0.05), and the treatment averages were compared using the Student–Newman–Keuls test (SNK), with the GraphPad Prism 9.2.0 software. Afterwards, all data were submitted to a one-way ANOVA, with the feeding substrate as the main effect. When significant differences were found among treatments, the Tukey–Kramer HSD test was used to compare the values obtained for different feeding substrates statistically. Means were compared to the Least Significant Difference (LSD) Test at a 5% probability level.

## 4. Results

### 4.1. Effect of Feeding Different Diets on Various Larval Parameters of T. molitor

**Figure 1:**
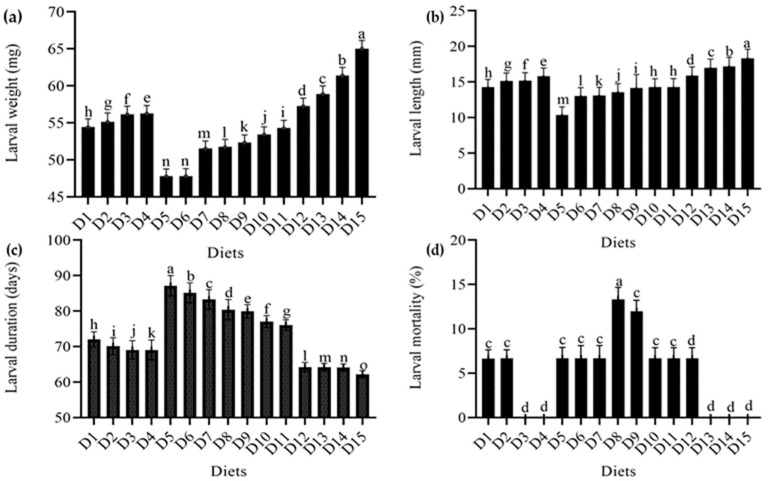
Effect of different diets on various larval parameters: (**a**) average weight (mg); (**b**) average length (mm); (**c**) average duration (days); (**d**) average mortality (%) of T. molitor under lab conditions. D1: ospor (2 mL); D2: ospor (4 mL); D3: ospor (6 mL); D4: ospor (8 mL); D5: imutec (0.2 g); D6: imutec (0.4 g); D7: imutec (0.6 g); D8: imutec (0.8 g); D9: fungi (250 g); D10: fungi (500 g); D11: fungi (750 g); D12: yeast (50 g); D13: yeast (100 g); D14: yeast (150 g); D15: wheat bran. Different letters above the bar graph shows significant different among the treatments.

When alternative meals were provided, there was a significant change in the *T. molitor* larval weight (DF = 9, F = 21163, *p* < 0.001). The maximum larval weight was 65.03 mg in the wheat bran standard diet and 61.40 mg in the wheat bran combination with yeast (*S. cerevisiae*) level (150 g). In wheat bran levels (0.2 g and 0.4 g) with imutec (*L. rhamnosus*), the minimum larval weight was 47.78 and 47.81 mg, respectively. When the level of these diets was raised, yeast (*S. cerevisiae*) and ospor (*B. clausii*) performed better, with more healthy larvae and greater larval weight, than imutec (*L. rhamnosus*) and fungi (*C. indica*). The larval weight recorded in yeast (*S. cerevisiae*) at different levels (50 g, 100 g, and 150 g) was 57.27 mg, 58.90 mg, and 61.40 mg, respectively, whereas in the case of the ospor (*B. clausii*) diet, larval weight was 54.43 mg, 55.14 mg, 56.15 mg, and 56.25 mg at different levels (2 mL, 4 mL, 6 mL, and 8 mL, respectively). The imutec (*L. rhamnosus*) diet produced lower larval weights of 47.78 mg, 47.81 mg, 51.54 mg, and 51.74 mg from diets containing 0.2 g, 0.4 g, 0.6 g, and 0.8 g of feed, respectively. The fungi (*C. indica*) diet produced significantly higher larval weights of 52.33 mg, 53.42 mg, and 54.29 mg from diets containing 250 g, 500 g, and 750 g of feed, respectively (Figure 1a). Moreover, the results of larval length demonstrate that varied diet combinations had a substantial effect on larval length (DF = 9, F = 25,826, *p* < 0.001). However, the greatest larval length was 18.32 mm in wheat bran (standard diet), followed by 150 g yeast (*S. cerevisiae*) at 17.19 mm, and the minimum larval length was 10.36 mm in imutec (*L. rhamnosus*) (0.2 g). The larval lengths recorded in diet combinations of imutec (*L. rhamnosus*) at levels of 0.2 g, 0.4 g, 0.6 g, and 0.8 g were 10.36 mm, 13.04 mm, 13.09 mm, and 13.58 mm, respectively. For the fungi (*C. indica*) diet at levels of 250 g, 500 g, and 750 g, the lengths obtained were 14.15 mm, 14.25 mm, and 14.25 mm, respectively. For the ospor (*B. clausii)* diet at levels of2 mL, 4 mL, 6 mL, and 8 mL, the lengths obtained were 14.25 mm, 15.14 mm, 15.18 mm, and 15.83 mm, respectively. For the yeast (*S. cerevisiae*) diet at levels of 50 g, 100 g, and 150 g, the lengths obtained were 15.89 mm, 16.97 mm, and 17.19 mm, respectively. Smaller larvae were produced by imutec (*L. rhamnosus*), fungi (*C. indica*), than by ospor (*B. clausii*) and yeast (*S. cerevisiae*). The results also suggest that ospor (*B. clausii*) and yeast (*S. cerevisiae*) levels increased larval length and vice versa (Figure 1b). Similarly, the highest larval duration in imutec (*L. rhamnosus*) at a level of 0.2 g was 87.12 days, while the minimum larval duration in the wheat bran control was 62.20 days. Maximum larval duration in imutec (*L. rhamnosus*) at levels of 0.2 g, 0.4 g, 0.6 g, and 0.8 g was 87.12 days, 85.12 days, 83.26 days, and 80.38 days, respectively. The minimum larval duration in yeast (*S. cerevisiae*) at levels of 50 g, 100 g, and 150 g was 64.20 days, 64.16 days, and 64.12 days, respectively (Figure 1c). However, the highest larval mortality rate was 13.33 percent in imutec (*L. rhamnosus*) at (0.8 g), while the lowest larval mortality rate was 6.6400 percent in ospor (*B. clausii*) at (2 mL). The results also suggest that ospor (*B. clausii*) and yeast (*S. cerevisiae*) had a lower larval mortality than imutec (*L. rhamnosus*) and fungi (*C. indica*). In terms of larval mortality, no larval mortality was seen in wheat bran (standard diet). It demonstrates that wheat bran was completely safe, with no negative effects on the larval stage of *T. molitor* (Figure 1d).

### 4.2. Effect of Different Diets on Different Pupal Parameters of T. molitor

**Figure 2:**
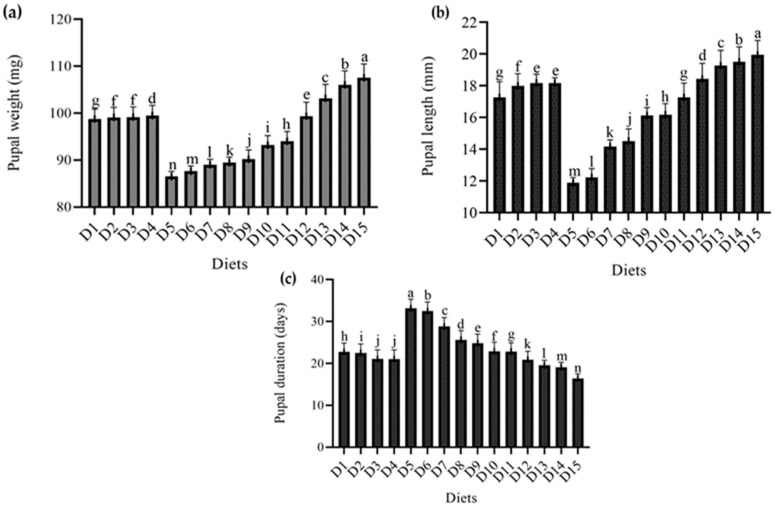
Effect of different diets on various Pupal parameters (**a**) pupal weight (mg), (**b**) pupal length (mm), (**c**) pupal duration (days), of *T. molitor* under lab conditions. D_1_: ospor (2 mL); D_2_: ospor (4 mL); D_3_: ospor (6 mL); D_4_: ospor (8 mL); D_5_: imutec (0.2 g); D_6_: imutec (0.4 g); D_7_: imutec (0.6 g); D_8_: imutec (0.8 g); D_9_: fungi (250 g); D_10_: fungi (500 g); D_11_: fungi (750 g); D_12_: yeast (50 g); D_13_: yeast (100 g); D_14_: yeast (150 g); D_15_: wheat bran. Different letters above the bar graph shows significant different among the treatments.

Figure 2 shows the results of the effect of several diets on pupal characteristics *T. molitor*. The results showed that varied diet combinations had a substantial effect on pupae growth, length, and duration, except for pupal mortality, which was zero in all diet combinations.

Maximum pupal weight was recorded in wheat bran (standard diet) at 107.55 mg, followed by wheat bran combination with yeast (*S. cerevisiae*) at levels of 150 g and 100 g, with pupal weights of 106.05 mg and 103.18 mg, respectively, and imutec (*L. rhamnosus*) being the least effective diet, having a minimum weight of 86.50 mg at 0.2 g. Pupae were significantly lighter in weight in the case of imutec (*L. rhamnosus*) and fungi (*C. indica*), with low weights of 86.50 mg, 87.66 mg, 89.02 mg, and 89.45 mg from feed levels of 0.2 g, 0.4 g, 0.6 g, and 0.8 g, respectively, and higher weights of 90.17 mg, 93.18 mg, and 94.02 mg from feed levels of 250 g, 500 g, and 750 g, respectively. Ospor (*B. clausii*) and yeast (*S. cerevisiae*) pupal weights ranged from 98.78 mg, 99.12 mg, 99.17 mg, and 99.50 mg from feed levels of 2 mL, 4 mL, 6 mL, and 8 mL, respectively, to 99.37 mg, 103.18 mg, and 106.05 mg from feed levels of 50 g, 100 g, and 150 g, respectively (Figure 2a). Similarly, a length of 19.94 mm was recorded in the case of wheat bran (standard diet). This was followed by yeast (*S. cerevisiae*) at levels of 50 g, 100 g, and 150 g, which produced larger size pupae of 19.52 mm, 19.26 mm, and 18.42 mm, respectively. In comparison, smaller pupae were observed in imutec (L. rhamnosus) at levels of 0.2 g, 0.4 g, 0.6 g, and 0.8 g, which produced pupae of 11.88 mm, 12.22 mm, 14.16 mm and 14.52 mm, respectively. Diets containing both imutec (*L. rhamnosus*) and fungi (*C. indica*) generated smaller pupae than others, including the conventional diet (Figure 2b). However, in terms of pupal duration, *T. molitor* spent the shortest time as a pupa on a standard diet (16.42 days), followed by yeast (*S. cerevisiae*) at levels of 50 g, 100 g, and 150 g, attaining durations of 20.91 days, 19.59 days, and 19.13 days, respectively. Imutec (*L. rhamnosus*) achieved the longest pupal duration, with levels 0.2 g, 0.4 g, 0.6 g, and 0.8 g lasting 33.17 days, 32.52 days, 28.81 days, and 25.66 days, respectiely. Imutec (*L. rhamnosus*) and fungi (*C. indica*) were also found to extend *T. molitor* pupa duration compared to other diets (Figure 2c). The results also show that varied diets influence pupal mortality. The findings show that the studied diets had no effect on pupal mortality. 

### 4.3. Effect of Feeding Different Diets on Various Adult Parameters of T. molitor

**Figure 3:**
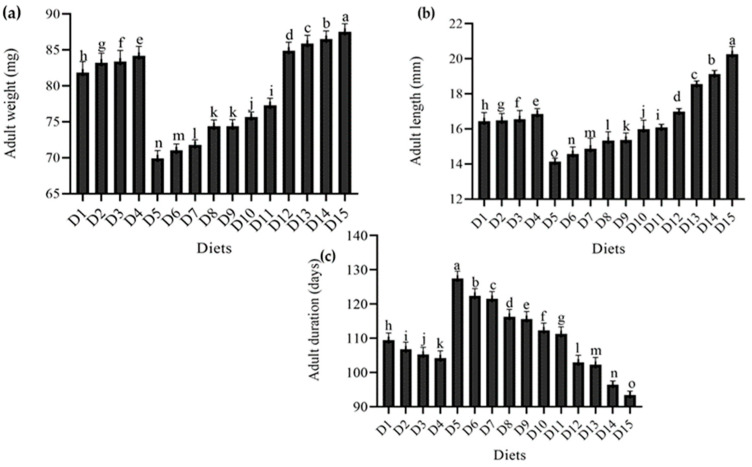
Effect of different diets on various adult parameters (**a**) adult weight (mg), (**b**) adult length (mm), (**c**) adult duration (days), of *T. molitor* under lab conditions. D_1_: ospor (2 mL); D_2_: ospor (4 mL); D_3_: ospor (6 mL); D_4_: ospor (8 mL); D_5_: imutec (0.2 g); D_6_: imutec (0.4 g); D_7_: imutec (0.6 g); D_8_: imutec (0.8 g); D_9_: fungi (250 g); D_10_: fungi (500 g); D_11_: fungi (750 g); D_12_: yeast (50 g); D_13_: yeast (100 g); D_14_: yeast (150 g); D_15_: wheat bran. Different letters above the bar graph shows significant different among the treatments.

The results of the current study regarding the impact of feeding various diets on different growth parameters of *T. molitor* are described in Figure 3. The results show that the diets evaluated had a substantial effect on adult metrices of *T. molitor*.

There was a significant range in adult weight. However, the greatest adult weight in the wheat bran (standard diet) group was 87.52 mg, followed by yeast (*S. cerevisiae*) at levels of (150 g) 86.50 mg, (100 g) 85.89 mg, and (50 g) 84.19 mg, and ospor (*B. clausii*) at levels of (8 mL) 84.17 mg, (6 mL) 83.39 mg, (4 mL) 83.24 mg, and (2 mL) 81.84 mg. The smallest adult weight was recorded in the imutec (*L. rhamnosus*) group, with feed levels of 0.8 g, 0.6 g, 0.4 g, and 0.2 g producing adult weights of 74.38 mg, 71.80 mg, 71.03 mg, and 69.90 mg, respectively. All diet combinations were better than imutec combined with wheat bran at different levels. Adult weights in the fungi (*C. indica*) group fed at levels of 250 g, 500 g, and 750 g were 74.41 mg, 75.68 mg, and 77.31 mg, respectively, which were substantially greater than imutec (*L. rhamnosus*) but significantly lower than ospor (*B. clausii*), yeast (*S. cerevisiae*), and wheat bran (standard diet) (Figure 3a). Similarly, a similar tendency was noted in adult length. The largest adult length (20.26 mm) was recorded in the wheat bran (standard diet) group, followed by the yeast (*S. cerevisiae*) group, growing to 19.12 mm, 18.55 mm, and 16.99 mm when fed at levels of 150 g, 100 g, and 50 g, respectively. The ospor (*B. clausii*) group grew to 16.86 mm, 16.55 mm, 16.49 mm, and 16.44 mm when fed at levels of 8 mL, 6 mL, 4 mL, and 2 mL, respectively. The minimum adult lengths in the imutec (*L. rhamnosus*) group were 14.14 mm, 14.57 mm, 14.87 mm, and 15.34 mm when fed at levels of 0.2 g, 0.4 g, 0.6 g, and 0.8 g, respectively. In general, larger beetles were produced by yeast (*S. cerevisiae*) and ospor (*B. clausii*) than by fungi (*C. indica*) and imutec (*L. rhamnosus*) (Figure 3b). However, different meal combinations had a substantial effect on adult longevity. Imutec (*L. rhamnosus*) fed at levels of 0.2 g, 0.4 g, 0.6 g, and 0.8 g produced the longest adult durations of 127.42 days, 122.38 days, 121.52 days, and 116.26 days, respectively. The yeast (*S. cerevisiae*) group had the shortest adult durations of 102.88 days, 102.22 days, and 96.40 days when fed at levels of 50 g, 100 g, and 150 g, respectively (Figure 3c).

### 4.4. Effect of Different Diets on Proximate Composition of T. molitor Larvae

**Figure 4:**
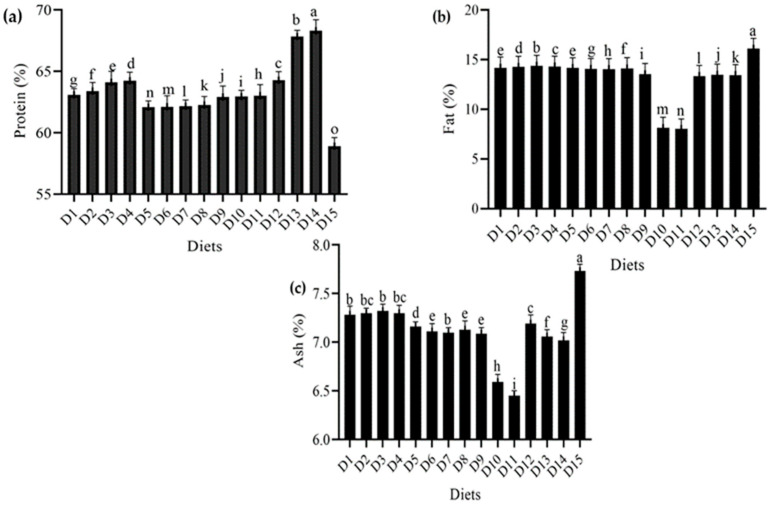
Effect of different diets on the proximate composition (**a**) protein %, (**b**) fat %, (**c**) ash % of *T. molitor* reared under lab conditions. D_1_: ospor (2 mL), D_2_: ospor (4 mL); D_3_: ospor (6 mL); D_4_: ospor (8 mL); D_5_: imutec (0.2 g); D_6_: imutec (0.4 g); D_7_: imutec (0.6 g); D_8_: imutec (0.8 g); D_9_: fungi (250 g); D_10_: fungi (500 g); D_11_: fungi (750 g); D_12_: yeast (50 g); D_13_: yeast (100 g); D_14_: yeast (150 g); D_15_: wheat bran. Different letters above the bar graph show significant different among the treatments.

The proximate composition (protein, fat, and ash contents) of dried *T. molitor* larvae raised on various meals is shown in Figure 4. *T. molitor’s* nutritional profile varied greatly depending on the food mix. 

As shown in Figure 4, different combinations of wheat bran were added to other diets, including ospor, imutec, fungi, and yeast. Protein content was found to be higher in yeast (*S. cerevisiae*)—with the levels of 150 g, 100 g, and 50 g containing 68.31 percent, 67.84 percent, and 64.28 percent, respectively—than in ospor (*B. clausii*)—with the levels of 8 mL, 6 mL, 4 mL, and 2 mL containing 64.23 percent, 64.11 percent, 63.39 percent, and 63.09 percent, respectively. A lower protein content was recorded in fungi (*C. indica*), with the feed levels of 750 g, 500 g, and 250 g containing 63.02 percent, 62.95 percent, and 62.91 percent, respectively. This was followed by imutec (*L. rhamnosus*), with the feed levels of 0.8 g, 0.6 g, 0.4 g, and 0.2 g containing 62.26 percent, 62.17 percent, 62.12 percent, and 62.09 percent protein, respectively. In comparison, a lower protein content was recorded in wheat bran (standard diet) at 58.91% (Figure 4a). Similarly, *T. molitor* fat content was measured in various feeding regimes that were considerably different. The highest fat content was recorded in the wheat bran control at 16.11 percent, whereas the lowest fat content was observed in the fungi (*C. indica*) level of 750 g, which had a fat content of 8.02 percent. *T. molitor* larvae fed on bacterial diets ospor (*B. clausii* and *L. rhamnosus*) showed higher fat content than fungus diets (*C. indica* and *S. cerevisiae*) (Figure 4b). However, *T. molitor’s* ash content varied significantly in response to different food combinations. Wheat bran (standard diet) had the highest ash level of 7.73 percent, whereas wheat bran combined with fungi (*C. indica*) at a level of 750 g had an ash content of 6.45% (Figure 4c).

## 5. Discussion

The larvae of mealworms are a popular pet food and a promising source of alternative protein-rich animal feed [5,20]. Mealworms have been proposed as a bio-regenerative life support system for space missions and are suitable for human nutrition, in addition to being used as animal feed [22,23]. Mealworms have a valuable protein profile and are simple to breed and feed. They are produced industrially as pet and zoo animal feed for birds, small mammals, batrachians, reptiles, and fish for these reasons. They are typically fed live but can also be purchased in powder, canned, or as dried food [22]. Although dried and canned larvae are commercially available, mealworms are typically fed live. Mealworms are a good substitute for traditional livestock feed [9]. Mealworms can replace or partially replace fish or soybean meal in poultry diet studies, and the results were comparable or even slightly superior in terms of growth and digestibility [9,23].

Global food system experts believe that the mealworm (*T. molitor*) could be a valuable source of food for humans [24,25,26]. Mealworms and insects generally have not drawn much attention outside of specialized social groups in terms of their value as a food source. However, they represent a cutting-edge nutrition source with clear advantages over traditional food types. Like many insect species, mealworms reproduce quickly, need little care, can be raised in confined spaces, and produce food with nutritional values comparable to beef, pork, and poultry. Despite these general traits, the literature on mealworm rearing and physiology does not include much research into the larvae’s ability to convert food into energy efficiently. According to the standards for food labels developed by the World Health Organization and Food and Agriculture Organization of the United Nations, mealworms’ nutritional components can be categorized as “high in” and a “source of” [27,28,29,30,31,32].

The yellow mealworms appear to be a valuable protein source for consumption by people. It is essential to create the technological infrastructure to enable the affordable mass production of insects for human consumption. The mealworms are large compared to other insects suggested for protein production and can, therefore, be harvested at an earlier stage of their development. The results indicate that the larvae must be harvested before they begin to prepare for the pupal stage, as they begin to lose weight at this time [33,34,35,36,37].

Mealworms’ various biological traits were significantly impacted by their various larval diets. The different nutritional values of the available larval diets may cause this variation in various biological parameters. Most of the components of wheat bran, which also include cellulose and hemicelluloses, starch (10–20%), lignin (4–8%), and protein (15–22%), are non-starch polysaccharides (46%) [38]. 

We employed mealworms (*T. molitor*) in this experiment, which was carried out in a controlled laboratory setting at a temperature of 25.5 ± 5 °C, a humidity of 60–65%, and a light/dark cycle of 10:14. According to Bordiean et al. [39], the insects were housed in a laboratory at a temperature of 28 degrees Celsius, a humidity of 55 to 60 percent, and a light cycle of 12 h. The only study known to have used low rearing temperatures (20 °C) was published in 1976 by Martin et al. [31]. They stated that temperatures beyond 35 °C are “unstable”, and that, at 40 °C, *T. molitor* will die. An ambient temperature of 25 °C to 27 °C is suggested for growing purposes. *T. molitor* larvae can be successfully raised in a rather dry environment (between 13% and 70% relative humidity). Similarly, Mellanby [40], found that, across a range of rearing humidity from 0% to 90%, the larvae exposed to higher humidity consistently retained more weight than the larvae exposed to lower humidity.

In this investigation, mealworms were discovered to have been provided weekly supplies of fresh potato slices as a source of moisture. Raw, fresh vegetables (such as potatoes, carrots, or lettuce) were also recommended, to provide the essential moisture to a feed medium consisting of wheat flour and wheat bran or oatmeal and maize bran [41,42]. High moisture content materials like potatoes can stimulate microbial development, and even cause the culture to disintegrate, as described by Ghaly and Alkoaik, [13].

Based on our findings, we found that feeding wheat bran solely to larvae was the most successful method, leading to the heaviest larvae (65.03 mg). Mealworms fed the WB 100, and WB 75 diets weighed in at 47.9 and 44.0 mg, respectively, as reported by Bordiean et al. [39].

According to the findings of several early researchers, wheat bran is the primary food material used for rearing mealworms in large numbers. The current study aimed to assess the efficacy of various supplements added to wheat bran for rearing *T. molitor*.

According to this study, after being exposed to (*C. indica)* fungus and (*B. clausii*) ospor (*C. indica*) is a fungus, *T. molitor* larvae grew faster and bigger. However, this fungus provides a wide range of vitamins and minerals to insects, as well as being the best source of protein for them. According to a similar study, Morales et al. [43], found that feeding insects a high-protein diet accelerated their development from egg to larva. 

Minimum larval weight was observed in the case of wheat bran levels with other diets. In contrast to what was found by Martin et al. [39], who found a range of 57 mg to 145 mg for the least and maximum larval weights, we found that the average larval weight was 91 mg. Our results contradict those of Hardouin and Mahoux [44], who found a minimum larval length of 28 mm and a maximum of 32 mm while rearing flies on a diet of poultry feed and urban bio-waste (catering to organic waste). Feeding wheat bran to mealworm larvae greatly reduced the time the insects spent in the larval stage. Our results supported the claim made by Weaver and Mcfarlane, [17], that the shortest possible lifespan of a mealworm was 62 days. Mortality was calculated as the number of larvae that did not survive until the experiment was finished. The results we obtained are supported by Greenberg and Ar [45]. Mortality rates for the larvae varied among diets, with wheat bran resulting in the lowest rates overall. Vitamin deficiency was found to increase larval mortality [37]. In future, it will be necessary to introduce dietary supplements to help decrease larval mortality.

It is possible that the extended pupal duration seen in fungi (*C. indica*) and conventional diet is due to the hardening of the pupal case, which delayed the pupae’s emergence. No studies reported the percentage of *T. molitor* pups that died on the studied diets. Unfortunately, this means that the results of the current study cannot be compared to those of previous studies. More research is needed to pinpoint the precise cause of pupal death [46,47,48].

Contrary to our results, Finke [22], indicated that the average adult body weight was 136 mg. Our results are consistent with those of Kim et al. [49], who found that mealworms fed wheat bran ranged in size from 15.5 to 16 mm long. Diets high in wheat bran were associated with dramatically reducing the adult lifespan. Our research confirmed the minimum length of 96 days provided by Ghaly et al. [13]. Life expectancy in adulthood was also recorded to be 94 days, as reported by Cotton [50].

Mealworms’ proximate composition was significantly impacted by the food they ate. Protein content ranged from 62.7% to 68.3% across all diets (58%). Wheat bran included notably more crude fat (16.11 percent) and ash (13.03%) than other diets (7.73%). The results of our investigation are consistent with those of a study on mealworms published by Ng et al. [6], which found 5.5% ash, 16.9% crude fat, and 63.6% crude protein, as stated by the authors (results on a dry matter basis). While our results for crude protein and crude fat were similar, those for ash were not. In contrast to our findings, Aguilar et al. [51], showed that *T. molitor* had a greater protein content (59.4%) but a far higher fat content (30.40%). Our findings agree with those of Barker et al. [52], who found that the crude protein levels of mealworms range from 45.9 to 68.9%.

Ghaly and Alkoaik [13] report that yellow mealworms’ protein, fat, and ash percentages range between 63.31% and 68.87%, 29.83% and 31.17%, and 4.3% and 5.7%, respectively. Although our results were similar to theirs in terms of crude protein, they were different in that they contained more fat and less ash.

Our findings differed significantly from those of the research mentioned above Siemianowska et al. [19]. Both the protein content (44.72%) and the ash content (3.69%) were discovered to be much lower than what was expected by the authors. While we identified a very low fat content, the same authors found a very high one (43.45%). The results from the study by Ravzanaadii et al. [53], found that the larval samples tested had a protein content of 46.44% and a fat content of 32.87%. The protein and fat contents were drastically different from our findings.

## 6. Conclusions

The study concluded that mealworm (*T. molitor*) development across all examined stages was considerably influenced by the mealworm’s overall nutrient profiles. When assessing larval, pupal, and adult weights and lengths, wheat bran (the conventional diet) was proven to be as successful as all of the other diets examined. The bacterial diet ospor (*B. clausii*) and the fungal diet yeast (*S. cerevisiae*) were discovered to be equally effective. The protein content of mealworms (*T. molitor*) on the fungal diet yeast (*S. cerevisiae*) and bacterial diet ospor was significantly greater than that of fungus (*C. indica*), imutec (*L. rhamnosus*) and wheat bran. Ospor (*B. clausii*) and yeast (*S. cerevisiae*) added to wheat bran, a staple food, enhanced various health indicators. Mealworms should be fed either a diet made up of wheat bran and yeast (*S. cerevisiae*) or wheat bran and ospor, for optimum growth (*B. clausii*).

## Data Availability

The datasets used and analyzed during the current study are available from the corresponding author upon reasonable request.

## References

[1] DeFoliart G.R. (1992). Insects as human food: Gene DeFoliart discusses some nutritional and economic aspects. Crop Prot..

[2] Liu C., Zhao J., Melton L., Shahidi F., Varelis P. (2019). Insects as a Novel Food. Encyclopedia of Food Chemistry.

[3] Hurd K.J., Shertukde S., Toia T., Trujillo A., Pérez R.L., Larom D.L., Love J.J., Liu C. (2019). The cultural importance of edible insects in Oaxaca, Mexico. Ann. Entomol. Soc. Am..

[4] Woolf E., Zhu Y., Emory K., Zhao J., Liu C. (2019). Willingness to consume insect-containing foods: A survey in the United States. LWT.

[5] Van Huis A., Van Itterbeeck J., Klunder H., Mertens E., Halloran A., Muir G., Vantomme P. (2013). Edible Insects: Future Prospects for Food and Feed Security.

[6] Ng W.K., Liew F.L., Ang L.P., Wong K.W. (2001). Potential of mealworm (*Tenebrio molitor*) as an alternative protein source in practical diets for African catfish, *Clarias gariepinus*. Aquac. Res..

[7] Rumpold B.A., Schlüter O.K. (2013). Nutritional composition and safety aspects of edible insects. Mol. Nutr. Food Res..

[8] Tang Q., Dai Y., Zhou B. (2012). Regulatory effects of Tenebrio molitor Linnaeus on immunological function in mice. Afr. J. Biotechnol..

[9] Makkar H.P., Tran G., Heuzé V., Ankers P. (2014). State-of-the-art on use of insects as animal feed. Anim. Feed. Sci. Technol..

[10] Melis R., Braca A., Mulas G., Sanna R., Spada S., Serra G., Fadda M.L., Roggio T., Uzzau S., Anedda R. (2018). Effect of freezing and drying processes on the molecular traits of edible yellow mealworm. Innov. Food Sci. Emerg. Technol..

[11] Ding M., Yang S., Ding J., Zhang Z., Zhao Y., Dai W., Wu W. (2023). Gut Microbiome Associating with Carbon and Nitrogen Metabolism during Biodegradation of Polyethene in Tenebrio larvae with Crop Residues as Co-Diets. Environ. Sci. Technol..

[12] Caparros Megido R., Sablon L., Geuens M., Brostaux Y., Alabi T., Blecker C., Drugmand D., Haubruge É., Francis F. (2014). Edible insects acceptance by B elgian consumers: Promising attitude for entomophagy development. J. Sens. Stud..

[13] Ghaly A.E., Alkoaik F. (2009). The yellow mealworm as a novel source of protein. Am. J. Agric. Biol. Sci..

[14] Siemianowska E., Kosewska A., Aljewicz M., Skibniewska K.A., Juszczak L.P., Jarocki A., Jedras M. (2013). Larvae of mealworm (*Tenebrio molitor* L.) as European novel food. Agri Sci..

[15] Choi H.-S., Kim S.-A., Shin H.-J. (2015). Present and perspective on insect biotechnology. KSBB J..

[16] Hu B., Das P., Lv X., Shi M., Aa J., Wang K., Wu X. (2022). Effects of ‘Healthy’ Fecal Microbiota Transplantation against the Deterioration of Depression in Fawn-Hooded Rats. mSystems.

[17] Weaver D.K., McFarlane J. (1990). The effect of larval density on growth and development of Tenebrio molitor. J. Insect Physiol..

[18] Damborsky M., Sandrigo-Ybran T., Oscherov E. (2000). Ciclo de Vida de Tenebrio molitor (Coleoptera, Tenebrionidae) en Condiciones Experimentales. http://www.unne.edu.ar/unnevieja/Web/cyt/cyt/biologia/b-011.pdf.

[19] Hill D.S. (2002). Pests of Stored Foodstuffs and Their Control.

[20] Fanatico A.C., Arsi K., Upadhyaya I., Morales-Ramos J., Donoghue D., Donoghue A.M. (2018). Sustainable fish and invertebrate meals for methionine and protein feeds in organic poultry production. J. Appl. Poult. Res..

[21] Kröncke N., Grebenteuch S., Keil C., Demtröder S., Kroh L., Thünemann A.F., Benning R., Haase H. (2019). Effect of different drying methods on nutrient quality of the yellow mealworm (*Tenebrio molitor* L.). Insects.

[22] Finke M.D. (2002). Complete nutrient composition of commercially raised invertebrates used as food for insectivores. Zoo. Biol..

[23] Sun J., Jia Q., Li Y., Zhang T., Chen J., Ren Y., Fu S. (2022). Effects of *Arbuscular Mycorrhizal* Fungi and Biochar on Growth, Nutrient Absorption, and Physiological Properties of Maize (*Zea mays* L.). J. Fungi.

[24] Yang K., Geng Q., Luo Y., Xie R., Sun T., Wang Z., Tian J. (2022). Dysfunction of FadA-cAMP signalling decreases *Aspergillus flavus* resistance to antimicrobial natural preservative Perillaldehyde and AFB1 biosynthesis. Environ. Microbiol..

[25] Pan C., Yang K., Erhunmwunsee F., Li Y., Liu M., Pan S., Tian J. (2023). Inhibitory effect of cinnamaldehyde on Fusarium solani and its application in postharvest preservation of sweet potato. Food Chem..

[26] Zhang Y., Zhang S., Yang X., Wang W., Liu X., Wang H., Zhang H. (2022). Enhancing the fermentation performance of frozen dough by ultrasonication: Effect of starch hierarchical structures. J. Cereal Sci..

[27] Wang Y., Liu S., Yang X., Zhang J., Zhang Y., Liu X., Wang H. (2022). Effect of germination on nutritional properties and quality attributes of glutinous rice flour and dumplings. J. Food. Compos. Anal..

[28] Li L., Xie B., Dong C., Hu D., Wang M., Liu G., Liu H. (2015). Rearing *Tenebrio molitor* L. (Coleptera: Tenebrionidae) in the “Lunar Palace 1” during a 105-day multi-crew closed integrative BLSS experiment. Life Sci. Space Res..

[29] Veldkamp T., Van Duinkerken G., van Huis A., Lakemond CM M., Ottevanger E., Bosch G., Van Boekel T. (2012). Insects as a Sustainable Feed Ingredient in Pig and Poultry Diets—A Feasibility Study.

[30] Bovera F., Loponte R., Marono S., Piccolo G., Parisi G., Iaconisi V., Gasco L., Nizza A. (2016). Use of larvae meal as protein source in broiler diet: Effect on growth performance, nutrient digestibility, and carcass and meat traits. J. Anim. Sci..

[31] Martin R.D., Rivers J.P.W., Cowgill U.M. (1976). Culturing mealworms as food for animals in captivity. Int. Zoo Yearb..

[32] Durst P.D., Shono K., Durst P.B., Johnson D.V., Leslie R.N., Shono K. (2010). Edible forest insects: Exploring new horizons and traditional practices. Forest Insects as Food: Humans Bite Back.

[33] Vantomme P., Mertens E., van Huis A., Klunder H. (2012). Assessing the Potential of Insects as Food and Feed in Assuring Food Security: Summary Report.

[34] Codex Alimentarius Commission (2007). Food Labelling.

[35] Nowak V., Persijn D., Rittenschober D., Charrondiere U. (2016). Review of food composition data for edible insects. Food Chem..

[36] Zhang F., Pant D., Logan B.E. (2011). Long-term performance of activated carbon air cathodes with different diffusion layer porosities in microbial fuel cells. Biosens. Bioelectron..

[37] Feng Q., Feng Z., Su X., Bai Y.D., Ding B.Y. (2021). Design and Simulation of Human Resource Allocation Model Based on Double-Cycle Neural Network. Comput. Intel. Neurosci..

[38] Gao J., Sun H., Han J., Sun Q., Zhong T. (2022). Research on Recognition Method of Electrical Components Based on FEYOLOv4-tiny. J. Elect. Engineer. Technol..

[39] Bordiean A., Krzyżaniak M., Stolarski M.J., Czachorowski S., Peni D. (2020). Will yellow mealworm become a source of safe proteins for Europe?. Agriculture.

[40] Mellanby K. (1932). The effect of atmospheric humidity on the metabolism of the fasting mealworm (*Tenebrio molitor* L., Coleoptera). Proc. R. Soc. Lond..

[41] Lyons W. (1991). Rearing Mealworms Fact Sheet.

[42] Gibson R. (2009). The Multipurpose Mealworm Leaping from the Box.com. http://www.leapingfromthebox.com/art/rlg/mealworms.html.

[43] Morales-Ramos J.A., Rojas M.G., Shapiro-llan D.I., Tedders W.L. (2013). Use of nutrient self-selection as a diet refining tool in *Tenebrio molitor* (Coleoptera: Tenebrionidae). J. Entomol. Sci..

[44] Hardouin J., Mahoux G. (2003). Zootechnie D’insects—Elevage et Utilization au Benefice de I’homme et de Certains Animaux.

[45] Greenberg S., Ar A. (1996). Effects of chronic hypoxia, normoxia, and hyperoxia on larval development in the beetle *Tenebrio molitor*. J. Insect Physiol..

[46] Fraenkel G. (1950). The nutrition of the mealworm, *Tenebrio molitor* L. (Tenebrionidae, Coleoptera). Physiol. Zool..

[47] Cheng M., Cui Y., Yan X., Zhang R., Wang J., Wang X. (2022). Effect of dual-modified cassava starches on intelligent packaging films containing red cabbage extracts. Food Hydrocoll..

[48] Yuan Q., Kato B., Fan K., Wang Y. (2023). Phased array guided wave propagation in curved plates. Mech. Syst. Signal Process..

[49] Kim S.Y., Park J.B., Lee Y.B., Yoon H.J., Lee K.Y., Kim N.J. (2015). Growth characteristics of mealworm (*Tenebrio molitor*). J. Sericultural Entomol. Sci..

[50] Cotton R.T. (1927). Notes on the biology of the meal worms, *Tenebrio molitor* Linne and *T. obscurus* Fab. Ann. Entomol. Soc. Am..

[51] Aguilar-Miranda E.D., Lopez M.G., Escamilla-Santana C., Rosa A.P. (2002). Characteristics of maize flour tortilla supplemented with ground *Tenebrio molitor* larvae. J. Agr. Food Chem..

[52] Barker D., Fitzpatrick M.P., Dierenfeld E.S. (1998). Nutrient compotion of selected whole invertebrates. Zoo Biol..

[53] Ravzanaadii N., Kim S.H., Choi W.H., Hong S.J., Kim N.J. (2012). Nutritional value of mealworm, *Tenebrio molitor* as food source. Int. J. Ind. Entomol..

